# Prediction of Fracture Loads in 3D-Printed ASA and Carbon-Fiber Reinforced ASA Notched Specimens Using the Calibrated ASED Criterion

**DOI:** 10.3390/ma18214966

**Published:** 2025-10-30

**Authors:** Sergio Arrieta, Sergio Cicero, José A. Álvarez

**Affiliations:** LADICIM, Departamento de Ciencia e Ingeniería del Terreno y de los Materiales, Universidad de Cantabria, Av. Los Castros, 44, 39005 Santander, Spainalvareja@unican.es (J.A.Á.)

**Keywords:** additive manufacturing, acrylonitrile-styrene-acrylate, notch effect, fracture, average strain energy density

## Abstract

This paper presents an adapted methodology for the prediction of fracture loads in additively manufactured (fused filament fabrication) polymers that exhibit non-linear behavior. The approach is based on the Average Strain Energy Density (ASED) criterion, which is typically limited to materials which develop fully linear-elastic behavior. Thus, in those cases where the material has a certain (non-negligible) amount of non-linear behavior, the ASED criterion needs to be corrected. To extend its applicability, this work proposes a thorough calibration of the ASED characteristic parameters: the critical value of the strain energy and the volume of the corresponding control volume. This enables the extrapolation of the linear-elastic formulation to non-linear situations. The approach is validated using acrylonitrile-styrene-acrylate (ASA) and 10 wt.% carbon-fiber reinforced ASA specimens. Single-edge-notched bending (SENB) specimens with three different raster orientations (0/90, 45/−45, and 30/−60) and four U-notch radii (0.0 mm—crack-like, 0.50 mm, 1.0 mm, and 2.0 mm) were printed and tested. The results demonstrate that the proposed calibration of the ASED criterion allows for accurate predictions of failure loads, providing a reliable tool for the structural integrity assessment of 3D-printed components.

## 1. Introduction

Additive manufacturing (AM) is a versatile fabrication technology capable of producing intricate geometries from diverse materials, including polymers, metals, ceramics, and composites [1]. This research focuses on fused filament fabrication (FFF), a prominent AM technique. FFF involves the extrusion of a molten filament through a heated nozzle, which is then deposited layer-by-layer onto a build platform to construct the final component [2].

Historically, FFF has been primarily utilized for rapid prototyping rather than for the production of load-bearing structural components. This is primarily due to the generally lower mechanical properties of 3D-printed components compared to those achieved via conventional methods, like injection molding [3]. Consequently, with the aim of improving the mechanical behavior of FFF-printed materials so that they can be used for structural applications, significant research efforts over the past years have aimed to clarify the correlation between FFF process parameters and the resulting mechanical performance of a wide range of thermoplastic polymers [4,5,6,7,8,9,10,11,12].

The complex geometries inherent to 3D-printed parts often contain stress risers, which can arise from manufacturing defects like poor surface finish and porosity, operational damage, or intrinsic design features such as grooves, holes, and corners. The response of the material to the presence of such stress raisers is a key factor the structural integrity of structural components. Assessing the corresponding structural integrity requires specific analytical approaches beyond conventional fracture mechanics (which addresses crack-like defects). It has been widely demonstrated (e.g., [13,14]) that non-sharp stress risers, generally referred to as notches, induce an apparent fracture toughness in the material that typically exceeds the intrinsic fracture toughness measured using cracked specimens. To effectively account for this notch effect, various methodologies have been developed, notably the Theory of Critical Distances [15] or the Average Strain Energy Density (ASED) criterion [14], on which this work is focused.

The ASED criterion has been extensively validated across a range of brittle and quasi-brittle materials under various loading conditions (e.g., [14,16,17,18,19]), including fatigue assessments [20,21,22,23,24]. This methodology has also been applied to analyze the fracture behavior of 3D-printed polymers [25,26,27,28,29,30]. However, to the best knowledge of the authors, it has not yet been applied specifically to 3D-printed acrylonitrile-styrene-acrylate, nor its carbon-fiber reinforced variant, both having moderate non-linear fracture behavior.

This study employs a relatively simple calibration technique to predict fracture loads using the ASED criterion, eliminating the need for complex, additional analyses. While this calibration approach has been successfully applied to conventional structural steel fracture mechanics specimens [19] and 3D-printed polymeric plates [27,31], this work analyzes its capacity to estimate fracture loads of single-edge-notched bend (SENB) specimens made of FFF-printed ASA and carbon-fiber reinforced ASA, extending the validation of the resulting analysis procedure. A total of 72 fracture specimens for each material were fabricated, with varying raster orientations and notch radii. Following experimental testing, the critical loads were measured and the ASED criterion was applied to estimate these values. This analysis was performed using both the standard linear-elastic ASED formulation and a specific calibration process that accounts for non-linear phenomena. The experimental and the theoretical values were then compared to evaluate the accuracy of the proposed methods.

The main objective, thus, has been to demonstrate that the calibrated ASED approach provides accurate predictions of fracture loads in 3D-printed polymers and 3D-printed polymer matrix composites with significant non-linear behavior, contributing to the future use of these types of materials in structural parts.

The paper is structured as follows: Section 2 details the materials, fabrication process, and experimental and numerical procedures; Section 3 presents the ASED predictions and their comparison with experimental data; and Section 4 summarizes the main findings and discusses the applicability of the ASED criterion for FFF-printed polymers.

## 2. Materials and Methods

Acrylonitrile-styrene-acrylate (ASA) is a high-performance thermoplastic widely used in FFF 3D printing, prized for its excellent mechanical properties and resistance to UV radiation and weathering [32,33,34,35]. It exhibits robust mechanical properties [36,37,38], including high impact strength [39], wear resistance [40], and durability [41]. Carbon-fiber reinforced ASA is an emerging material that significantly enhances the mechanical properties of the base polymer, such as tensile strength and stiffness [42,43,44]. The resistance of ASA to environmental degradation makes it particularly well-suited for outdoor components, in addition to its use in prototyping [44,45,46,47]. Thus, ASA is a very good alternative to the widely used acrylonitrile-butadiene-styrene (ABS). Although their compositions and mechanical properties are similar, ASA is more durable in harsh environments [35,48]. Additionally, since ASA is generally reported to have moderate mechanical properties, this research also includes carbon-fiber reinforced ASA (10 wt.%, ASA-CF10), with the aim of exploring how the introduction of carbon fibers affects the corresponding mechanical performance.

Specimens of ASA and ASA-CF10 were fabricated via FFF in a CreatBot F430 printer (Henan Suwei Electronic Technology Co., Ltd., Zhengzhou, China). The following parameters were used: layer height of 0.2 mm, line width of 0.42 mm, 100% infill density, printing temperature of 250 °C, bed temperature of 90 °C, and a printing speed of 40 mm/s. These parameters were selected based on the manufacturer’s recommendations.

**Figure 1:**
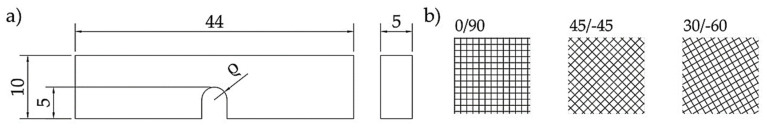
(**a**) Schematic of fracture SENB specimens. Nominal dimensions (in mm), with ρ being 0.0 mm, 0.5 mm, 1.0 mm, and 2.0 mm; and (**b**) raster orientations.

A detailed description of the experimental program of ASA [49] and ASA-CF10 [50] was recently published by the authors. To sum up, a total of 162 tests were carried out, consisting of 9 tensile tests and 72 fracture (SENB) tests for each material. All specimens were fabricated using additive manufacturing in a flat print orientation. The fracture specimens incorporated four distinct notch radii, indicated as ρ in Figure 1: 0.0 mm (simulating crack-like defects, made by sawing with a razor blade), 0.5 mm, 1.0 mm, and 2.0 mm. Additionally, three different raster orientations (ROs) (0/90, 45/−45, and 30/−60) were investigated for the fracture specimens. Figure 1 provides a schematic of the specimen geometry and the distinct raster orientations employed.

**Figure 2:**
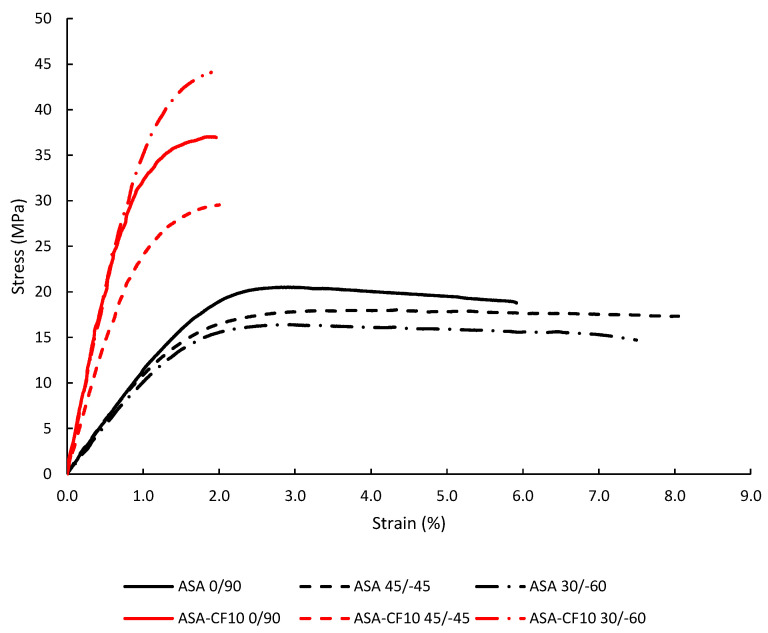
Tensile curves obtained for ASA and ASA-CF10.

In these studies, tensile tests followed ASTM D638 [51], while fracture tests on both cracked and notched specimens observed ASTM D6068 [52]. The key mechanical properties derived from these tests are presented in Table 1 [49,50]. As shown in Figure 2, the stress-strain curves reveal that the ASA-CF10 material behaves much closer to a linear-elastic material compared to pure ASA.

After determining the fundamental mechanical properties from the tensile and fracture tests, the failure loads on the U-notched specimens were predicted by applying the ASED criterion. The initial approach utilized the original formulation, which assumes a linear-elastic material behavior. This step established a baseline for the fracture load predictions and provided a direct point of comparison to the subsequent analysis, which incorporates the non-linear behavior of the material.

The ASED criterion, based on the “core region” concept introduced by Sih [53] and subsequently refined by Lazzarin and Zambardi [54,55], has found extensive application in the prediction of material fracture processes. The ASED approach posits that brittle fracture under tensile stress initiates when the mean elastic strain energy density (W) within a defined control area attains a critical value (W_C_). For an ideally brittle material, the critical strain energy density can be determined as described in [54]:

(1) $$W_{C}=\frac{{\sigma_{u}}^{2}}{2E}$$

The ultimate tensile strength (σ_u_) and Young’s modulus (E) are critical parameters in material characterization. W_C_ results, using average values of σ_u_, are gatherer in Table 2. The ASED method involves calculating the total area beneath the non-linear tensile stress–strain curve, rather than relying on a simplified linear approximation of Equation (1). This modified ASED approach will be adopted in the present study to account for the observed non-linear material behavior.

The ASED criterion necessitates the calculation of the strain energy density averaged over a defined control volume circumscribing the notch tip. For two-dimensional (plane) analyses, this control volume is represented as a circle or a circular sector with a characteristic radius, R_C_. In instances where the notch opening angle (2α) is zero, characteristic of U-notches, R_C_ can be quantitatively determined using the fracture toughness (K_mat_) of the material, ultimate tensile strength, and Poisson’s ratio (ν) [56].

(2) $$R_{C}=\frac{\left(1+\upsilon \right)\left(5-8\upsilon \right)}{4\pi }{\left(\frac{K_{mat}}{\sigma_{u}}\right)}^{2}\;\;\;Plane\;strain,$$

(3) $$R_{C}=\frac{\left(5-3\upsilon \right)}{4\pi }{\left(\frac{K_{mat}}{\sigma_{u}}\right)}^{2}\;\;\;Plane\;stress,$$

**Figure 3:**
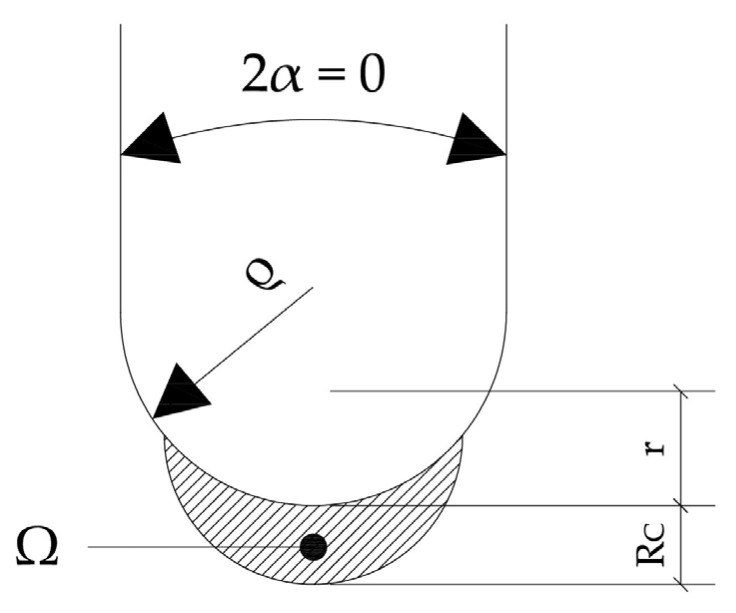
Control area for U-notch under Mode I loading.

As supported by the findings of [15], when the fracture toughness reaches the limit imposed by Equation (4), plane strain conditions dominate. Meanwhile, when fracture toughness exceeds the limit defined by Equation (5), the plane stress conditions are reached.

(4) $$K_{mat}<\sigma_{y}\cdot {\left(\frac{B}{2.5}\right)}^{1/2}\;\;\;Plane\;strain,$$

(5) $$K_{mat}>\sigma_{y}\cdot {\left(\pi B\right)}^{1/2}\;\;\;Plane\;stress,$$

where B represents the specimen thickness and σ_y_ denotes the yield strength. For blunt notches, the total strain energy is quantifiable within the crescent-shaped volume (Figure 3). Subsequently, the ASED can be formulated as a function of the maximum elastic notch stress (σ_max_) [57]. The total strain energy over a given area (Ω) is quantified by the ASED. It can be determined using Equation (6), as described in [14]:

(6) $$\bar{W}=F\left(2\alpha \right)H\left(2\alpha ,\frac{R_{C}}{\rho }\right)\frac{{\sigma_{max}}^{2}}{E}$$

The parameter F(2α) is contingent upon the notch opening angle, with a value of 0.785 for U-notches (2α = 0). H depends on both the notch geometry (opening angle, 2α, and notch radius, ρ) and the material properties (critical radius, R_C_). σ_max_ represents the maximum elastic stress at the notch tip. Table 3 provides a compilation of H function values for U-shaped notches [58] for a material with a Poisson’s ratio of 0.30 [49,50].

The fracture behavior, particularly the critical load, will be investigated using the ASED criterion, whose critical condition is established as follows:

(7) $$\bar{W}=W_{C}$$

The corresponding critical value, W_C_, is derived from the entire area under the material tensile curve, rather than from Equation (1). By combining Equations (6) and (7), the following relationship is established:

(8) $$\sigma_{max-ASED}=\sqrt{\frac{W_{C}\cdot E}{0.785\cdot H\left(2\alpha =0,\frac{R_{C}}{\rho }\right)}}$$

The derivation of the H-function necessitates the precise definition of the material critical radius. This parameter is determined by comparing and selecting between two distinct methodologies, represented by Equations (2) and (3). For the materials and specimens examined in this study, plane stress conditions are achieved. Therefore, Equation (3) is the appropriate relation for calculating R_C_.

**Figure 4:**
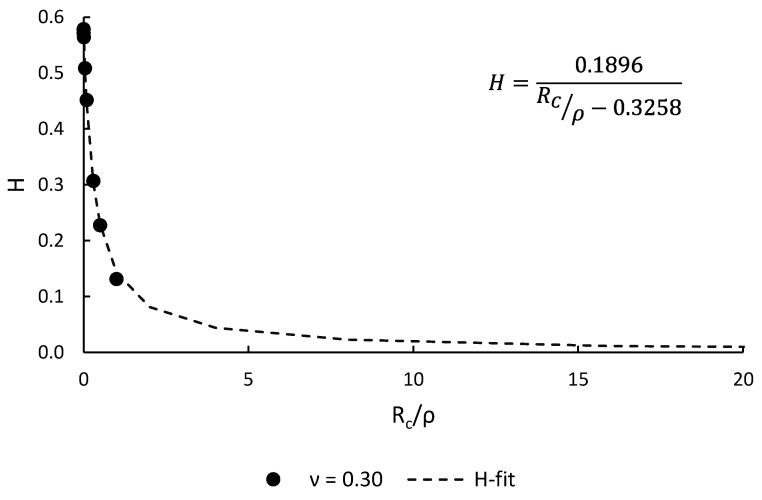
Fitting equation used to derive H values for R_C_/ρ > 1.0 (data from [58]).

During the analysis, the ratio of the critical radius to the notch radius (R_C_/ρ) exceeded unity. This necessitated the extrapolation of H values [16], tabulated in Table 3, utilizing the fitting function presented in Figure 4. This extrapolation was critical to ensure the accuracy and completeness of the analytical results.

However, as demonstrated by previous studies [9,59], the materials under investigation exhibit noticeable non-linear tensile behavior across three distinct raster orientations. This observed non-linearity indicates that the assumption of a purely linear-elastic response, upon which the ASED criterion is typically founded, is not appropriate for an accurate characterization of this material. Despite this non-linearity, if its extent is limited, alternative approaches for applying the ASED criterion have been proposed [17], and validated for AM polymers [25,26,27,28,31].

**Figure 5:**
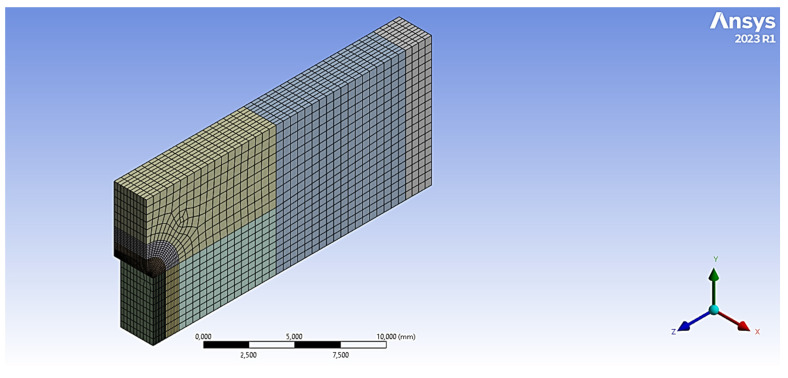
FE model of the SENB specimen (ρ = 0.5 mm).

The determination of the external load corresponding to the maximum stress at a notch tip can be achieved through numerical or analytical methods. Finite element simulations offer a robust numerical approach for this derivation. The stress field within the mid-plane of the fracture section (at the notch tip) was analyzed for all specimen geometries using a FE model developed in Ansys (2023 R1) [27]. A quarter-model approach, exploiting the symmetry inherent in fracture tests, was employed (Figure 5), assuming linear-elastic material behavior (material properties from Table 1). A refined mesh was implemented in the vicinity of the notch tip due to the high sensitivity of stress concentration to mesh size in this region. For every nominal notch radius, the mesh sizes were 61,435 nodes and 13,620 elements for ρ = 0.5 mm; 59,124 nodes and 13,090 elements for ρ = 1.0 mm; and 70,316 nodes and 15,670 elements for ρ = 2.0 mm.

**Figure 6:**
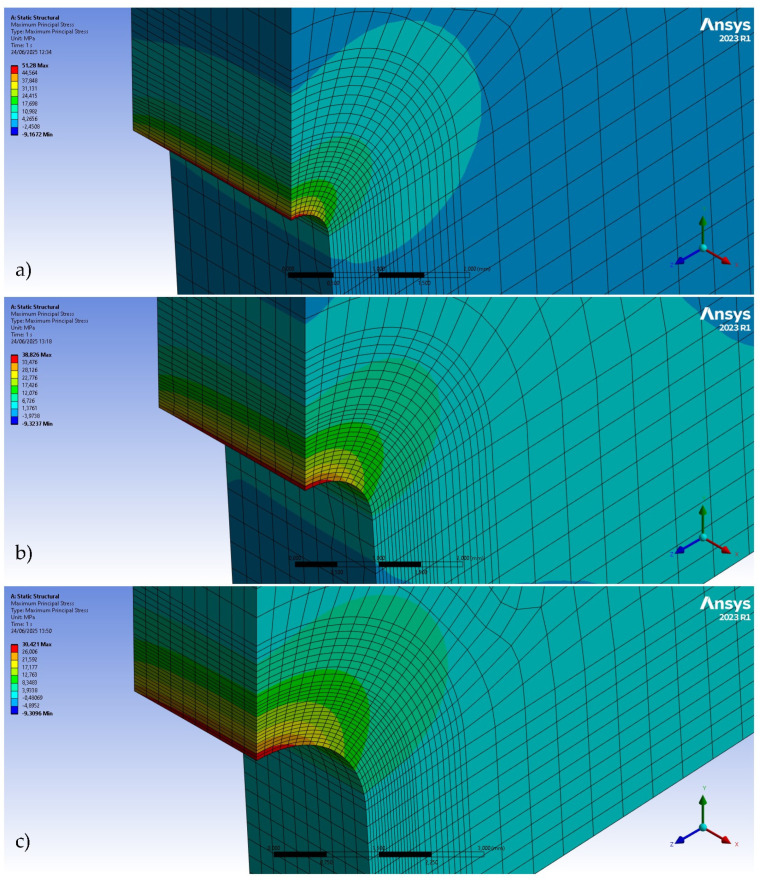
FE model for U-notched specimens: (**a**) ρ = 0.5 mm; (**b**) ρ = 1.0 mm; and (**c**) ρ = 2.0 mm. Applied load P_FE_ = 10 N.

The maximum principal stress at the notch tip (σ_max-FE_) was calculated for an arbitrary external load (P_FE_ = 10 N), as shown in Figure 6. Subsequently, the critical load was determined through proportionality, as defined by Equation (9). Taking into account the potential for the stress field to be highly sensitive to specimen geometry, particularly the notch radius, and acknowledging that actual geometrical parameters may deviate from nominal values, a unique simulation was conducted for each specimen type in pursuit of analysis efficiency. The σ_max-ASED_ was calculated using Equation (8).

(9) $$P_{\text{ASED-FE}}=P_{\mathrm{FE}}\frac{\sigma_{\text{max-ASED}}}{\sigma_{\text{max-FE}}}$$

The experimental fracture loads (P_max_) and predictions by ASED criterion (P_ASED_) are gathered in Table A1 and Table A2 (Appendix A). As discussed in Section 3, the fracture load predictions for both ASA and ASA-CF10 are largely non-conservative, with few results becoming conservative.

When the material under investigation exhibits non-linear behavior, the standard ASED criterion, which assumes linear-elasticity, cannot be directly applied. To address this, the criterion requires a calibration of its characteristic parameters: the critical strain energy density (W_C_*) and the control radius (R_C_*) [19,27,31,60]. This process effectively creates an “equivalent” linear-elastic material, allowing the standard ASED formulation to be used. The calibration in this study is based on the fracture loads of conventional SENB specimens with varying U-notch radii. The fracture loads from two distinct conditions (crack-like and 2.0 mm notched specimens) were used to establish the following relationships, based on the corresponding maximum stresses (σ_max1_ and σ_max2_) at the notch tip:

(10) $$F\left(2\alpha \right)H_{1}\left(2\alpha ,\frac{R_{C}}{\rho }\right)\frac{{\sigma_{max1}}^{2}}{E}=W_{C}$$

(11) $$F\left(2\alpha \right)H_{2}\left(2\alpha ,\frac{R_{C}}{\rho }\right)\frac{{\sigma_{max2}}^{2}}{E}=W_{C}$$

**Figure 7:**
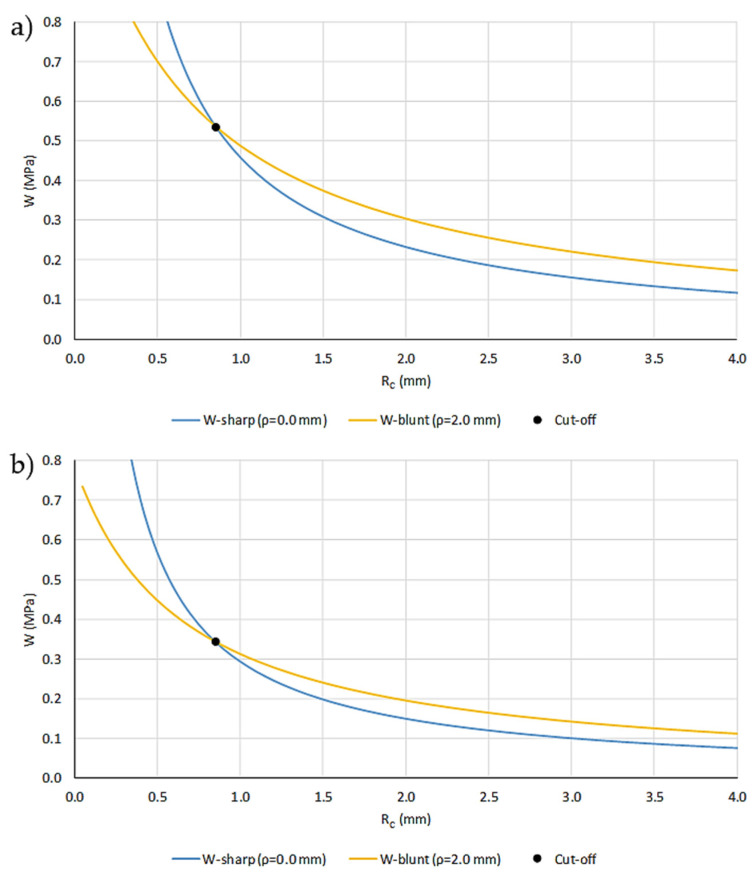
Example of calibration of W_C_* and R_C_* parameters with SENB U-notched specimens: (**a**) ASA 0/90; and (**b**) ASA-CF10 0/90.

By substituting different values of R_C_* into Equations (10) and (11), two distinct curves of W_C_* versus R_C_* are generated, as shown in Figure 7a and Figure 7b, for ASA 0/90 and ASA-CF10 0/90, respectively. The intersection point of these two curves provides the calibrated material parameters, W_C_* and R_C_*, which account for the non-linear behavior of the materials. The calibrated parameters are collected in Table 4.

Once the calibrated parameters have been successfully defined, the prediction of fracture loads (P_ASED_*) for any other notch radius becomes straightforward. This is achieved by utilizing the standard linear-elastic ASED procedure in conjunction with the calibrated parameters. Based on the ASED failure criterion, the maximum (critical) stress at the notch tip (σ_max-ASED_*) can be directly determined from Equations (1), (6), (7) and (8), using W_C_* and R_C_* instead of standard linear-elastic parameters (W_C_ and R_C_, in Table 2) calculated by the usual procedure. Finally, the critical load (P_ASED_*) was determined through proportionality, as defined by Equation (12).

(12) $$P_{ASED}^{*}=P_{\mathrm{FE}}\frac{\sigma_{\text{max-ASED}}^{*}}{\sigma_{\text{max-FE}}}$$

## 3. Results and Discussion

**Figure 8:**
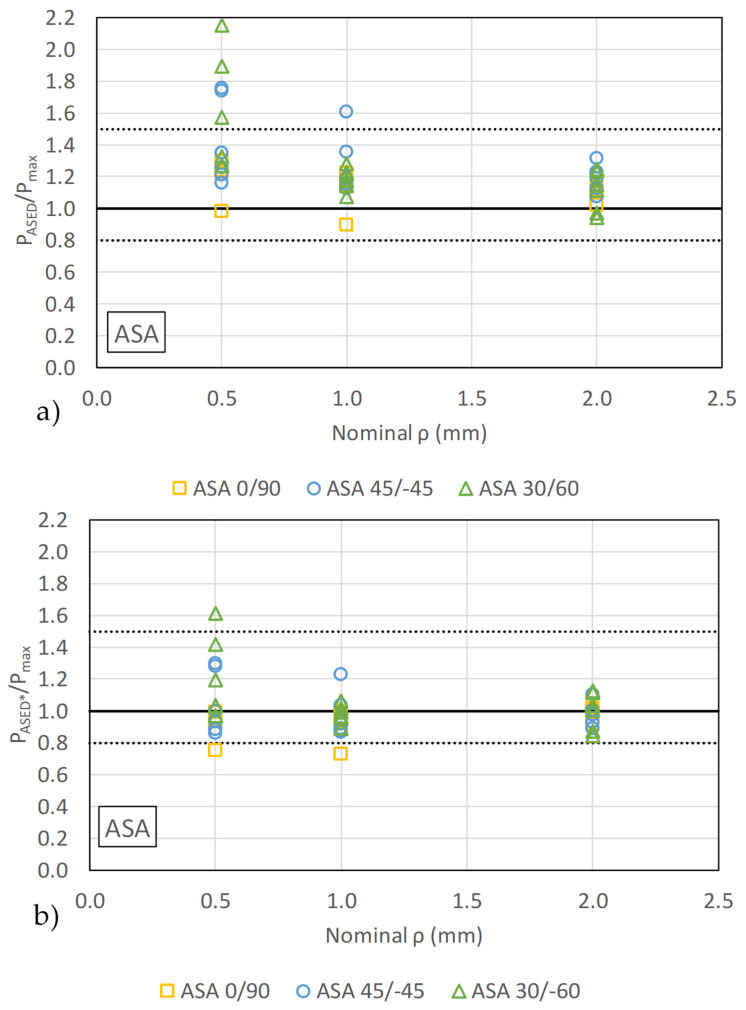
Comparison between experimental results and predictions for ASA by (**a**) standard linear-elastic ASED criterion (P_ASED_) and (**b**) calibrated ASED criterion (P_ASED_*).

**Figure 9:**
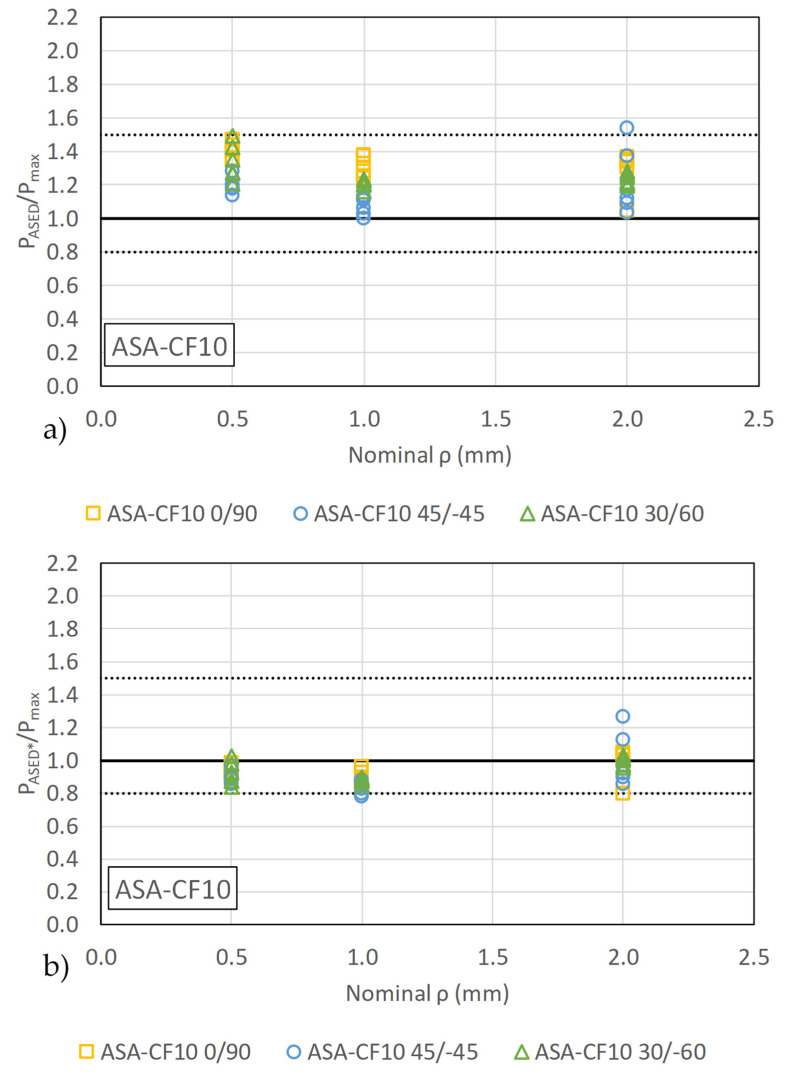
Comparison between experimental results and predictions for ASA-CF10 by (**a**) standard linear-elastic ASED criterion (P_ASED_) and (**b**) calibrated ASED criterion (P_ASED_*).

To establish a baseline for evaluating the suggested calibration technique, fracture load estimations (P_ASED_) were first derived by directly employing the standard linear-elastic ASED criterion. A comparison between these initial predictions and the actual experimental fracture loads (P_max_) is presented in Figure 8 and Figure 9 (with the corresponding data being gathered in Table A1 and Table A2). The results show that the fracture load estimations for the pure ASA material are significantly higher than the experimental values. Specifically, a mean ratio of P_ASED_/P_max_ of 1.24 highlights the poor predictive accuracy of the traditional approach. This considerable discrepancy is attributed to the inherent mismatch between the linear-elastic assumption of the standard ASED criterion and the clear non-linear (plastic) behavior exhibited by the ASA in both tensile and fracture tests.

The mean ratio P_ASED_/P_max_ for ASA-CF10 is 1.25 fall, but with lower scatter than ASA results, with less than a +50% margin compared to the experimental loads. This higher predictive accuracy is consistent with the mechanical response of the material, as the tensile test results shown in Figure 2 indicate a more limited degree of non-linearity compared to the pristine ASA.

The fracture predictions by standard ASED criterion (shown in Figure 8a for ASA, and in Figure 9a for ASA-CF10) are non-conservatives in general, with few results becoming conservative. P_ASED_/P_max_ ratios exhibit comparable values for different notch radii, except for ASA 45/−45 and 30/−60 with a notch radius of 0.5 mm, in which the precision is lower.

As described above, the linear-elastic ASED criterion provides limited accuracy when applied to the pristine ASA due to its significant non-linear behavior. To address this, we employed the calibrated ASED criterion technique detailed earlier (the resulting W_C_* and Rc* are presented in Table 4). The data clearly show that the calibrated critical mean strain energy density substantially deviates from the linear-elastic critical value proposed in the original ASED procedure. This deviation is particularly pronounced for the ASA material (W_C_ is multiplied by a factor of 3), quantitatively underscoring the necessity of this calibration to account for its inherent plasticity.

The application of the calibrated ASED criterion resulted in a significant improvement in predictive accuracy, particularly for materials exhibiting non-linear behavior. As illustrated in Figure 8b and Figure 9b (data summarized in Table A1 and Table A2), the predictions for the notched ASA and ASE-CF10 specimens became more accurate. Overall, the mean P_ASED_*/P_max_ ratios are now 1.00 and 0.93 for ASA and ASA-CF10, respectively, demonstrating that the calibrated ASED criterion provides better estimations of the fracture loads. It also significantly reduces the overestimation of predicted loads.

## 4. Conclusions

This research successfully applied the Average Strain Energy Density criterion to estimate fracture loads in FFF-printed ASA and carbon-fiber reinforced ASA (ASA-CF10) U-notched SENB specimens. Specimens were fabricated using three raster orientations (0/90, 45/−45, and 30/−60) and combining different notch radii (0.0 mm—crack-like defects, 0.50 mm, 1.0 mm, and 2.0 mm).

The conventional linear-elastic ASED criterion proved non-conservative for both materials. The predictions of fracture loads (P_ASED_) were considerably higher than the experimental results (P_max_), evidenced by P_ASED_/P_max_ ratios consistently above unit. The overestimation of critical loads can lead to unsafe structural analyses. The predictions for the ASA-CF10 specimens were better than those for the ASA, with a scatter of +50%. This outcome is attributed to the limited non-linearity of the composite material, which better adheres to the linear-elastic assumption.

To account for the non-linearity in ASA and ASA-CF10 materials, a calibration of the ASED parameters (W_C_* and R_C_*) was performed using experimental fracture loads from U-notched SENB specimens with ρ = 0.0 mm and ρ = 2.0 mm. This calibration noticeably improved the estimations for both ASA and ASA-CF10. The resulting average P_ASED_*/P_max_ ratios were 1.00 for ASA and 0.93 for ASA-CF10, demonstrating a high overall accuracy. Furthermore, the calibrated ASED criterion generally provides conservative predictions (i.e., P_ASED_* < P_max_), providing a safe condition for structural assessment.

The results confirm that the ASED criterion is a powerful methodology for providing accurate predictions of critical loads for notched components made of FFF-printed ASA and ASA-CF10. Crucially, achieving high accuracy and safe conservatism for materials with non-fully linear behavior requires a prior calibration process to correctly account for the non-linear material response.

This research validates the use of the calibrated ASED approach to estimate the critical loads of ASA and ASA-CF10 components containing notches. The analysis should be extended to different matrices and reinforcements in order to achieve a more complete and reliable validation of the estimates provided by the calibrated ASED criterion in 3D-printed polymeric materials with non-linear behavior.

## Figures and Tables

**Table 1:** Mechanical properties for FFF-printed ASA and ASA-CF10. E: Young’s modulus; σ_y_: yield stress; σ_u_: ultimate tensile strength; ε_u_: strain under maximum load; K_mat_: fracture toughness.

Material	RO	E (MPa)	σ_y_ (MPa)	σ_u_ (MPa)	ε_u_ (%)	K_mat_ (MPam^1/2^)
ASA [49]	0/90	1050 ± 66	15.47 ± 2.11	19.39 ± 0.99	2.8 ± 0.2	2.47 ± 0.19
	45/−45	1053 ± 22	14.13 ± 0.32	18.50 ± 0.72	4.5 ± 0.2	2.90 ± 0.23
	30–60	990 ± 28	12.32 ± 0.40	16.52 ± 0.11	2.8 ± 0.1	2.72 ± 0.22
ASA-CF10 [50]	0/90	4002 ± 236	32.18 ± 1.29	37.52 ± 0.41	1.8 ± 0.2	4.47 ± 0.17
	45/−45	2797 ± 174	25.57 ± 1.59	30.58 ± 1.81	2.0 ± 0.1	4.05 ± 0.20
	30–60	3496 ± 457	34.65 ± 5.71	39.66 ± 6.29	1.9 ± 0.1	4.46 ± 0.36

**Table 2:** Critical linear-elastic strain energy density values (W_C_).

Material	RO	W_C_ (MPa)
ASA	0/90	0.179
	45/−45	0.163
	30/−60	0.138
ASA-CF10	0/90	0.176
	45/−45	0.167
	30/−60	0.225

**Table 3:** Values of the function H for U-shaped notches in a material with υ = 0.30 [58].

2α (rad)	R_C_/ρ	H
0	0.0005	0.5785
0	0.001	0.5777
0	0.005	0.5714
0	0.01	0.5638
0	0.05	0.5086
0	0.1	0.4518
0	1	0.1314

**Table 4:** Calibrated ASED parameters: W_C_* and R_C_*.

Material	RO	W_C_* (MPa)	R_C_* (mm)
ASA	0/90	0.536	0.85
	45/−45	0.448	1.35
	30/−60	0.586	1.05
ASA-CF10	0/90	0.343	0.85
	45/−45	0.334	1.40
	30/−60	0.484	0.70

## Data Availability

The original contributions presented in this study are included in the article. Further inquiries can be directed to the corresponding author.

## Abbreviations

The following abbreviations are used in this manuscript:

ABS	Acrylonitrile-butadiene-styrene
AM	Additive manufacturing
ASA	Acrylonitrile-styrene-acrylate
ASA-CF10	Carbon-fiber reinforced ASA (10 wt.%)
ASED	Average Strain Energy Density criterion
ASTM	American Society for Testing and Materials
FE	Finite element
FFF	Fused Filament Fabrication
RO	Raster orientation
SENB	Single-edge-notched bending
TCD	Theory of Critical Distances

## Appendix A

This appendix compiles the experimental fracture loads (P_max_), the fracture load predictions obtained using the standard linear-elastic ASED criterion (P_ASED_), and those obtained using the calibrated ASED criterion (P_ASED_*) for ASA (Table A1) and ASA-CF10 (Table A2).

**Table A1:** Experimental and predicted critical loads for ASA SENB specimens.

RO	Test nº	ρ (mm)	W (mm)	B (mm)	a_0_ (mm)	P_max_ (N)	P_ASED_ (N)	P_ASED_/P_max_	P_ASED_* (N)	P_ASED_*/P_max_
0/90	1	0.00	9.86	5.18	4.75	101.6	-	-	-	-
	2	0.00	9.72	5.16	4.80	67.4	-	-	-	-
	3	0.00	9.55	5.00	4.78	78.0	-	-	-	-
	4	0.00	9.64	5.01	4.90	52.5	-	-	-	-
	5	0.00	9.75	5.13	4.80	66.2	-	-	-	-
	6	0.00	9.74	4.99	4.18	69.8	-	-	-	-
	1	0.51	9.67	5.09	5.17	71.9	88.43	1.231	67.73	0.943
	2	0.52	9.57	5.07	5.16	70.7	87.60	1.239	67.19	0.950
	3	0.50	9.40	5.05	5.15	72.8	89.28	1.227	68.30	0.938
	4	0.49	9.73	5.10	4.99	91.7	90.16	0.983	68.88	0.751
	5	0.50	9.64	5.00	4.90	68.7	89.28	1.300	68.30	0.994
	6	0.59	9.51	5.25	4.14	67.4	82.42	1.222	63.78	0.946
	1	1.03	9.74	4.99	5.13	68.5	83.51	1.220	68.00	0.993
	2	1.00	9.71	5.01	5.06	72.1	84.68	1.175	68.72	0.953
	3	1.01	9.83	4.95	5.06	74.2	84.29	1.136	68.48	0.923
	4	1.02	9.75	5.04	5.19	93.4	83.90	0.898	68.23	0.731
	5	1.01	9.70	5.02	5.11	73.4	84.29	1.149	68.48	0.934
	6	1.01	9.60	5.02	5.18	-	-	-	-	-
	1	2.10	9.70	5.03	4.37	69.3	77.04	1.111	69.14	0.998
	2	2.11	9.63	5.05	4.37	69.0	76.88	1.114	69.05	1.000
	3	2.12	9.67	5.10	4.36	69.9	76.72	1.098	68.96	0.987
	4	2.10	9.72	5.06	4.30	69.3	77.04	1.111	69.14	0.998
	5	2.13	9.60	5.01	4.34	75.1	76.56	1.020	68.87	0.917
	6	2.10	9.69	5.12	4.35	67.9	77.04	1.135	69.14	1.019
45/−45	1	0.00	9.74	5.00	4.94	68.5	-	-	-	-
	2	0.00	9.80	5.06	4.40	88.9	-	-	-	-
	3	0.00	9.84	5.14	5.07	87.8	-	-	-	-
	4	0.00	9.52	5.05	4.97	82.5	-	-	-	-
	5	0.00	9.58	5.10	5.02	82.0	-	-	-	-
	6	0.00	9.68	5.16	5.25	89.1	-	-	-	-
	1	0.60	9.14	5.39	4.77	54.3	95.38	1.755	70.51	1.297
	2	0.50	9.59	5.12	4.90	86.1	104.26	1.210	76.42	0.887
	3	0.70	9.67	5.01	4.77	76.4	88.48	1.158	65.96	0.863
	4	0.67	9.68	5.05	5.06	71.8	90.39	1.259	67.21	0.936
	5	0.60	9.36	5.20	5.10	70.5	95.38	1.351	70.51	0.999
	6	0.56	9.70	5.16	5.19	56.7	98.64	1.738	72.67	1.281
	1	1.13	9.57	5.04	4.58	78.0	92.80	1.189	71.55	0.917
	2	1.02	9.45	5.24	4.80	72.0	97.46	1.352	74.52	1.034
	3	1.10	9.52	5.23	4.71	82.9	94.00	1.133	72.31	0.872
	4	1.05	9.26	5.10	4.99	59.8	96.12	1.607	73.66	1.231
	5	1.10	9.64	5.14	5.08	83.6	94.00	1.124	72.31	0.865
	6	1.12	9.55	5.06	4.54	80.6	93.20	1.155	71.80	0.890
	1	2.14	9.71	5.02	5.28	81.5	87.83	1.076	72.43	0.888
	2	2.05	9.56	5.10	5.20	75.3	89.57	1.189	73.47	0.975
	3	2.02	9.54	5.12	5.40	73.3	90.18	1.229	73.84	1.006
	4	2.40	9.51	5.13	5.50	63.3	83.35	1.315	69.80	1.101
	5	2.20	9.50	5.12	4.52	77.0	86.72	1.126	71.78	0.932
	6	2.07	9.57	5.06	5.21	73.3	89.18	1.216	73.23	0.999
30/−60	1	0.00	9.76	5.14	4.66	79.8	-	-	-	-
	2	0.00	9.67	5.18	4.99	97.5	-	-	-	-
	3	0.00	9.73	5.13	5.02	66.6	-	-	-	-
	4	0.00	9.64	5.21	5.16	85.5	-	-	-	-
	5	0.00	9.67	5.11	4.86	71.9	-	-	-	-
	6	0.00	9.85	5.14	5.17	87.8	-	-	-	-
	1	0.40	9.64	5.13	4.58	86.0	109.02	1.267	83.65	0.972
	2	0.21	9.51	5.11	4.85	79.2	149.91	1.893	112.38	1.419
	3	0.22	9.47	5.15	4.85	68.2	146.49	2.149	109.95	1.613
	4	0.31	9.59	5.24	5.14	78.7	123.62	1.571	93.83	1.192
	5	0.40	9.70	5.14	5.24	86.0	109.02	1.268	83.65	0.973
	6	0.50	9.59	5.18	5.25	73.5	97.69	1.328	75.84	1.031
	1	1.13	9.66	5.23	4.55	67.9	86.85	1.279	72.05	1.061
	2	1.10	9.41	5.22	4.76	71.7	87.98	1.226	72.77	1.014
	3	1.00	9.45	5.23	4.69	75.7	92.10	1.216	75.44	0.996
	4	1.15	9.38	5.17	4.98	75.2	86.13	1.145	71.59	0.952
	5	1.12	9.60	5.23	5.38	81.2	87.22	1.074	72.29	0.890
	6	1.05	9.57	5.22	4.59	76.5	89.97	1.175	74.05	0.967
	1	2.12	9.66	5.06	4.29	66.2	82.41	1.244	74.38	1.123
	2	2.13	9.63	5.13	4.68	84.8	82.23	0.970	74.27	0.876
	3	2.11	9.60	5.08	4.43	74.2	82.59	1.112	74.49	1.003
	4	2.10	9.75	5.24	4.21	88.0	82.77	0.940	74.59	0.848
	5	2.05	9.67	5.25	4.37	72.8	83.70	1.149	75.14	1.031
	6	2.12	9.56	5.07	4.70	66.6	82.41	1.236	74.38	1.115

**Table A2:** Experimental and predicted critical loads for ASA-CF10 SENB specimens.

RO	Test nº	ρ (mm)	W (mm)	B (mm)	a_0_ (mm)	P_max_ (N)	P_ASED_ (N)	P_ASED_/P_max_	P_ASED_* (N)	P_ASED_*/P_max_
0/90	1	0.00	10.04	4.94	5.37	104.9	-	-	-	-
	2	0.00	10.11	4.97	5.77	86.1	-	-	-	-
	3	0.00	10.02	4.93	5.15	108.9	-	-	-	-
	4	0.00	10.15	5.00	5.62	101.7	-	-	-	-
	5	0.00	10.12	4.93	5.00	127.0	-	-	-	-
	6	0.00	10.14	4.95	4.63	-	-	-	-	-
	1	0.65	10.03	4.97	5.43	100.9	142.79	1.415	95.79	0.949
	2	0.65	10.10	5.05	5.55	99.6	142.78	1.433	95.78	0.961
	3	0.64	10.10	4.90	5.50	97.9	143.84	1.469	96.38	0.984
	4	0.65	10.11	4.97	5.50	101.6	142.78	1.405	95.78	0.943
	5	0.65	10.03	4.98	5.43	105.6	142.78	1.352	95.78	0.907
	6	0.66	10.20	4.98	5.64	105.5	141.75	1.344	95.20	0.903
	1	1.08	10.26	4.95	5.14	116.2	148.51	1.278	104.42	0.898
	2	1.09	10.12	4.98	5.32	119.4	147.88	1.239	104.08	0.872
	3	1.07	10.19	4.95	5.26	108.7	149.15	1.372	104.76	0.964
	4	1.07	10.24	4.99	5.30	112.7	149.15	1.324	104.76	0.930
	5	1.00	10.09	5.00	5.17	111.6	153.91	1.379	107.33	0.962
	6	1.06	10.24	4.92	5.43	120.2	149.80	1.246	105.11	0.874
	1	2.08	10.05	4.99	5.62	135.3	141.19	1.044	108.27	0.800
	2	2.14	10.25	4.98	5.75	103.4	139.47	1.349	107.42	1.039
	3	2.14	10.05	4.91	5.63	104.2	139.47	1.338	107.42	1.031
	4	2.14	10.03	4.94	5.59	102.0	139.47	1.368	107.42	1.053
	5	2.12	10.08	4.97	5.68	103.6	140.03	1.352	107.70	1.039
	6	2.10	10.20	4.97	5.72	107.8	140.61	1.304	107.98	1.002
45/−45	1	0.00	10.19	4.96	5.55	97.45	-	-	-	-
	2	0.00	10.31	4.9	5.44	110.12	-	-	-	-
	3	0.00	9.99	4.78	5.12	120.51	-	-	-	-
	4	0.00	10.12	4.94	5.16	114.78	-	-	-	-
	5	0.00	10.24	4.83	5.33	121.88	-	-	-	-
	6	0.00	10.24	4.87	4.80	150.37	-	-	-	-
	1	0.66	9.97	4.83	5.48	99.95	127.86	1.279	96.75	0.968
	2	0.66	10.19	4.92	5.60	108.37	127.86	1.180	96.75	0.893
	3	0.65	10.15	4.95	5.51	113.48	128.80	1.135	97.39	0.858
	4	0.64	10.32	4.9	5.72	108.05	129.76	1.201	98.05	0.907
	5	0.67	10.03	4.84	5.56	108.07	126.94	1.175	96.12	0.889
	6	0.64	10.06	4.87	5.47	108.15	129.76	1.200	98.05	0.907
	1	1.11	10.24	5.03	5.21	128.33	131.90	1.028	102.91	0.802
	2	1.15	10.21	4.96	5.37	129.45	129.73	1.002	101.47	0.784
	3	1.12	9.97	4.82	5.03	118.33	131.34	1.110	102.54	0.867
	4	1.12	10.22	5.03	5.41	116.22	131.34	1.130	102.54	0.882
	5	1.13	10.07	4.88	5.23	117.03	130.80	1.118	102.18	0.873
	6	1.13	10.23	4.93	5.42	123.25	130.80	1.061	102.18	0.829
	1	2.06	10.14	4.9	5.71	113.27	126.83	1.120	104.54	0.923
	2	2.07	10.15	4.91	5.66	116.17	126.56	1.089	104.37	0.898
	3	2.09	10.04	4.85	5.50	121.61	126.02	1.036	104.03	0.855
	4	2.07	9.99	4.68	5.81	92.42	126.56	1.369	104.37	1.129
	5	2.09	10.17	3.57	5.73	81.98	126.02	1.537	104.03	1.269
	6	2.08	10.24	4.77	5.77	107.59	126.29	1.174	104.20	0.968
30/−60	1	0.00	10.11	4.91	5.32	119.75	-	-	-	-
	2	0.00	10.22	4.87	5.69	107.36	-	-	-	-
	3	0.00	10.23	4.87	5.24	128.85	-	-	-	-
	4	0.00	10.01	4.9	4.94	135.00	-	-	-	-
	5	0.00	9.97	4.87	5.72	83.67	-	-	-	-
	6	0.00	10.13	4.89	5.11	112.86	-	-	-	-
	1	0.70	10.09	4.94	5.29	114.4	137.94	1.206	95.74	0.837
	2	0.64	10.17	4.89	5.40	106.5	143.92	1.351	99.06	0.930
	3	0.66	10.30	4.90	5.59	111.7	141.83	1.270	97.90	0.877
	4	0.64	9.99	4.86	5.39	96.3	143.71	1.493	99.06	1.029
	5	0.65	10.23	4.92	5.55	112.3	142.86	1.272	98.48	0.877
	6	0.66	9.99	4.89	5.20	99.6	141.54	1.421	97.90	0.983
	1	1.10	10.04	4.97	5.16	120.92	147.60	1.221	107.72	0.891
	2	1.10	10.19	4.90	5.40	122.94	147.60	1.201	107.72	0.876
	3	1.07	10.24	4.88	5.57	121.02	149.49	1.235	108.71	0.898
	4	1.11	10.14	4.91	5.11	122.12	146.99	1.204	107.40	0.879
	5	1.15	10.07	4.88	5.09	120.72	144.63	1.198	106.16	0.879
	6	1.13	10.16	4.91	5.41	125.41	145.80	1.163	106.77	0.851
	1	2.07	10.30	4.91	5.81	119.34	142.31	1.193	114.21	0.957
	2	2.12	10.05	4.90	5.59	109.84	140.87	1.283	113.52	1.034
	3	2.08	10.20	4.92	5.70	116.76	142.02	1.216	114.07	0.977
	4	2.09	10.13	4.92	5.62	112.43	141.73	1.261	113.93	1.013
	5	2.09	10.18	4.91	5.68	113.68	141.73	1.247	113.93	1.002
	6	2.10	10.35	4.92	5.85	117.07	141.44	1.208	113.79	0.972
